# Comparative study of the therapeutic efficacy of autologous platelet-rich plasma and honey in healing skin wounds in sheep

**DOI:** 10.14202/vetworld.2021.2170-2177

**Published:** 2021-08-23

**Authors:** Daikh Badis, Deffa Ouafa

**Affiliations:** 1 Department of Biology of Organisms, University of Batna 2, Batna, Algeria; 2 Biotechnology’s Laboratory of the Bioactive Molecules and the Cellular Physiopathology, University of Batna 2, Batna, Algeria; 3 Laboratory of Biology and Environment, Faculty of Nature and Life Sciences, University of Mentouri Brothers, Constantine, Algeria

**Keywords:** honey, platelet-rich plasma, sheep, skin, wound healing

## Abstract

**Background and Aim:**

This investigation is the continuation of a published preliminary study examining the therapeutic efficacy of platelet-rich plasma (PRP) as a topical treatment for skin wounds in sheep. The study aimed to compare the healing effects of autologous PRP with that of natural honey.

**Materials and Methods:**

This study involved nine clinically healthy male sheep. After sterile skin preparation, full-thickness longitudinal incision wounds were created on the backs of each animal. The animals were randomly divided into three groups of three sheep each. In Group I, the wounds were treated with PRP; in Group II, the wounds were treated with honey; and in Group III, the wounds were treated with saline solution. The different treatments were administered topically every 3 days. Healing was assessed by a semi-quantitative histopathological study from biopsies taken on the 3^rd^, 7^th^, 14^th^, 21^st^, and 28^th^ days of healing. The data obtained were compared using the non-parametric Mann–Whitney U-test, and p<0.05 and 0.01 were used to determine the level of significance of the recorded differences.

**Results:**

Semi-quantitative histopathological evaluation showed significant differences in the progression of wound healing between the three study groups. Recorded data showed that PRP may reduce inflammation during the first 3 days after the incision. Moreover, the synthesis and organization of collagen fibers were significantly improved in the group treated with PRP compared with those in the group treated with honey.

**Conclusion:**

PRP offers a promising therapeutic option for healing skin wounds in sheep compared with honey.

## Introduction

The skin forms a mechanically flexible barrier protecting higher organisms from various traumas and infections [1]. Restoring the structural integrity of the skin depends on the interplay of healing stages that begin immediately after the trauma. Successful healing involves an orderly orchestration of several processes: Cell migration, proliferation, and differentiation [2,3]. These empirical stages include inflammation, reepithelization, granulation tissue formation, wound contraction, and tissue remodeling. The last step corresponds to the formation of the extracellular matrix (ECM), which requires a sequence of events at the molecular, cellular, and tissue levels, including cell-cell and cell-matrix interactions [4,5]. Although fibroblasts and keratinocytes play a key role in wound healing, at the molecular level, other elements, such as growth factors, cytokines and their receptors, and matrix molecules, are essential [4].

Wound care during the healing process is particularly important to prevent complications, such as infections and keloids, which can compromise the esthetic aspect of healing [5]. One of the goals of modern medical science is to try to heal a wound in a shorter time with fewer side effects. In this context, many studies have been published, and various therapeutic agents that can affect and promote wound healing were proposed. Dohan *et al*. [6] have considered a new therapeutic approach for wound healing using autologous platelet concentrates (i.e., platelet-rich plasma [PRP]) derived from fibrin glue technology in the 1990s. Several publications on its effectiveness have been reported, but it remains widely controversial because of the absence of a consensus to standardize the protocol for its preparation [6,7]. Most animal experiments have highlighted that PRP accelerates wound healing and stimulates cell proliferation and granulation tissue formation. In contrast, for thousands of years, honey was known for its therapeutic wound healing properties. Long used in the 20^th^ century, it was gradually abandoned after World War II in favor of more modern, more innovative products, such as asiaticoside. Honey is recognized for its antibacterial activity and promotes wound healing through different mechanisms. It stimulates the proliferation of fibroblasts, improves the formation of granulation tissue, and decreases the activity of collagenase.

The increased interest in complementary therapies for skin healing, such as the use of synthetic or natural remedies, has led to a deep investigation of these products, but few studies have compared their modalities of action [8,9,10]. In this context, we conducted this study to evaluate the effectiveness of PRP and honey in healing skin wounds in sheep and compare their biological effects.

## Materials and Methods

### Ethical approval

This study was approved by the Ethics Committee of the Institute of Agronomic and Veterinary Sciences of the University of BATNA 1 – Algeria.

### Study period and location

The study was conducted from March to May 2019, on sheep belonging to the University of Batna 1, Algeria. During the experiment, all surgical maneuvers were performed in a laboratory at an ambient temperature of 25°C.

### Experimental animals

Nine healthy adult male sheep weighing 20-25 kg were used in this study. These animals were bred in the animal house of the Institute of Veterinary and Agronomic Sciences, University of Batna 1. All these animals had free access to water and an ordinary diet (i.e., straw and barley). One month before surgery, all sheep were dewormed using ivermectin at a dose of 0.2 mL/kg administered subcutaneously.

### Preparation of PRP and honey

The steps for preparing the PRP were performed according to a double centrifugation protocol proposed by Carneiro *et al*. [11] and modified by Daikh and Benoune [7]. Then, 20 mL whole blood was taken by a septic puncture of the jugular vein of each sheep. Then, the collected blood was dispensed into 5 mL tubes containing citrate dextrose acid. The speed of the first centrifugation was set at 1800 rpm for 8 min, and the second centrifugation was performed at 1000 rpm for 8 min. The total amount of PRP collected was 2.5 mL. All steps were performed at an ambient temperature of 22°C. For honey, it was harvested from the nectar of jujube plants in late spring in the mountainous region of Arris in Algeria. For isolating impurities, honey was passed through a Whatman 0.5 mm filter at 25-30°C, then transferred to a dark lid dish, and used in the research laboratory.

### The count of platelets and realization of smears

The platelet count was taken using a hemocytometer (Neubauer cell improved). Smears of whole blood and PRP were stained with May–Grünwald–Giemsa stain on each count to isolate the animals affected by hematological diseases and assess the richness of thrombocytes in the PRP.

### Experimental grouping of animals

The animals were randomized into three groups consisting of three sheep in each group. Various treatments were administered topically once on 3-day intervals. PRP was prepared on each administration and activated by adding calcium chloride (0.1 mL CaCl_2_ to each 1 mL PRP) immediately before its application [12]. For natural honey, 1 g was used directly on the wound surface.

·Group I: Animals treated with PRP
·Group II: Animals treated with honey

Group III: Animals treated with saline solution (NaCl 09%).

### Realization of skin wounds

Before all surgical interventions, the animals were first tranquilized with acepromazine at a dose of 0.1 mg/kg intramuscularly. Then, the skin was shaved and disinfected by applying an iodine solution of povidone (dermal betadine 10%). On the back of each animal, two full-thickness longitudinal incision wounds (7 cm in length) were created on each lateral side near the vertebral line [13]. The incision was made under the effect of local anesthesia (lidocaine hydrochloride 02%), induced by subcutaneous infiltration at a flow rate of 1 mL/1 cm^3^ [14]. Then, all wounds were sutured using a non-absorbable thread (STERIM*, T-30 mm-4/8). All rules of asepsis were strictly observed.

### Clinical follow-up

Every day, all laboratory animals were examined (in terms of animal behavior, body temperature, and cardiorespiratory activity, among others). Within the wounds, a daily macroscopic follow-up was performed throughout the experimentation period.

### Follow-up of healing: Semi-quantitative histopathological study

Skin samples using a biopsy punch (4 mm BP-40F) from the lesion incision sites were taken on the 3^rd^, 7^th^, 14^th^, 21^st^, and 28^th^ days after surgery. The samples were processed by routine histological procedures: First, they were fixed with buffered formalin, then embedded in paraffin solution, and cross-sectioned using a microtome into 4 μm thin sections. The tissue sections were stained with hematoxylin and eosin and examined for possible histopathological changes. The histopathological scores established by Vidinsky *et al*. [15] and Kilík *et al*. [16] were used in this study (presented in Table-1 by codes from 0 to 3).

**Table-1:** Significance of the different histopathological scores of the semi-quantitative histological evaluation of skin wound healing.

Score	Reepithelization step	PMNL	Macrophages	Fibroblasts	New blood vessels	New collagen
0	Thickening of cut edges	Absence	Absence	Absence	Absence	Absence
1	Migration of épithélial cells	Minimum	Minimum	Minimum	Minimum	Minimum
2	Bridging of the incision	Moderate	Moderate	Moderate	Moderate	Moderate
3	Complete régeneration	Marked	Marked	Marked	Marked	Marked

*Polymorphonuclear leukocytes. PMNLs=Polymorphonuclear leukocytes

### Statistical analysis

For each variable in the study, a descriptive statistical analysis was performed. Mean values±standard deviations were calculated. The data obtained from the semi-quantitative evaluation of the histopathological parameters were compared using the non-parametric Mann–Whitney U-test. All statistical analyses were performed using the Statistical Package for the Social Sciences, version 23.0. p<0.05 and 0.01 were used to determine the level of significance of the recorded differences.

## Results

### Clinical follow-up

After the surgical incisions were made, the animals remained healthy without clinical evidence of infection. All animals well tolerated the anesthesia and surgical procedure and did not appear to be affected by the presence of the wounds on their backs.

### PRP

**Figure-1:**
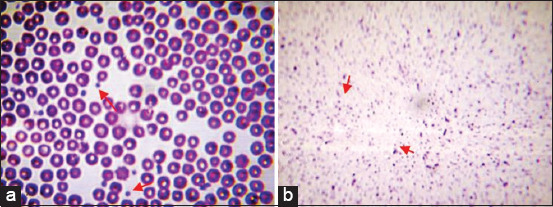
Smears showing thrombocytes (arrows) of whole blood (a), platelet-rich plasma (b). Stain: May-Grunwald-Giemsa (M.G.Gx100).

The mean platelet count is much higher in PRP (1463.2±95.22×10^3^/μL) than in whole blood (466.46±80.22×10^3^/μL). It is approximately 3-fold higher than that in whole blood (Figure-1).

### Histopathological follow-up

Semi-quantitative histopathological evaluation (Table-2) showed differences in the progression of healing in the three study groups.

**Table-2:** Results of the semi-quantitative analysis of the histopathological evaluation of the healing of skin wounds. Each calculated parameter was presented by its mean and standard deviation (mean±SD).

Day	Group	Reepithelization step	PMNL	Macrophages	Fibroblasts	New blood vessels	New collagen
Day 3	I	0.7±0.48	2.16±0.57−	1.25±0.45	0.83±0.71	0.75±0.45	0.33±0.49
	II	0.5±0.52	1.41±0.66	1.41±0.51	0.66±0.49	0.58±0.51	0.16±0.38
	III	0.2±0.42	1.25±0.45	1.58±0.51	0.33±0.49	0±0	0±0
Day 7	I	1.66±0.49−	2.5±0.67	1.08±0.28	1.91±0.66−	1.41±0.51	1.58±0.51
	II	1.41±0.51	2.16±0.71	1.5±0.52	1.58±0.66	1.16±0.38	1.41±0.51
	III	0.83±0.57	1.66±0.77	1.66±0.42−	1.08±0.79	1.25±0.45	1.16±0.57
Day 14	I	2.0±0.6	0.58±0.66	1.25±0.45	2.75±0.45−	2.90±0.31	2.75±0.45−−
	II	1.66±0.65	0.83±0.93	1.41±0.51	2.5±0.52	2.60±0.51	2.66±0.49−
	III	1.58±0.51	1.08±1.44	1.5±0.51	2.16±0.57	2.50±0.52	2.0±0.60
Day 21	I	2.75±0.45−	0.16±0.38	0.58±0.51	3.0±0	3.0±0	3.0±0
	II	2.25±0.62−	0.33±0.49	0.83±0.38	2.85±0.38	2.87±0.35	2.75±0.45
	III	1.91±0.79	0.58±0.66	1.00±0.42	2.66±0.49	2.44±0.52	2.5±0.52
Day 28	I	3±0	0.0±0	0.0±0	1.83±0.38	1.9±0.31	3.0±0
	II	2.75±0.45	0.0±0	0.0±0	1.66±0.49	2.33±0.50	3.0±0
	III	2.5±0.52	0.33±0.49	0.5±0.67	1.75±0.75	2.37±0.51	2.72±0.46

PMNL=Polymorphonuclear leukocytes

Three days after surgery, our results showed a significant difference in favor of PMNL, recorded in the group of animals treated with PRP (2.16±0.57).

**Figure-2:**
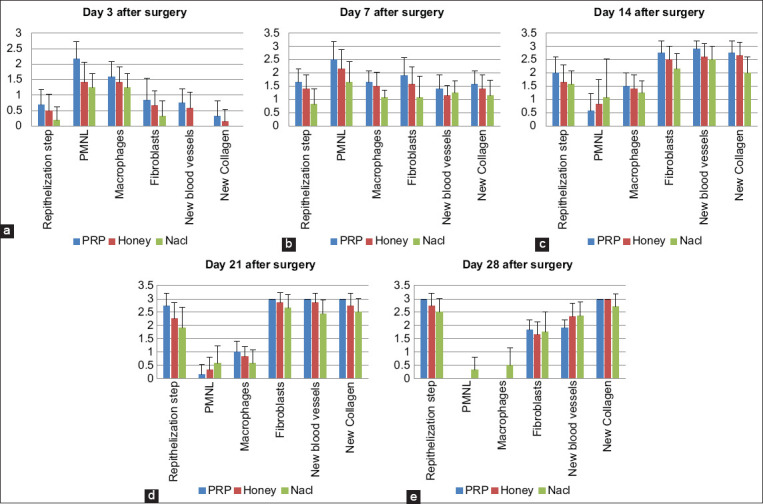
Results of the semi-quantitative analysis of the histopathological scores in the different groups of animals. (a) Results of the semi-quantitative analysis after 3 days, (b) after 7 days of healing, (c) after 14 days, (d) after 21 days. and (e) after 28 days of healing (data are presented as mean±SD; *p<0.05, **p<0.01).

**Figure-3:**
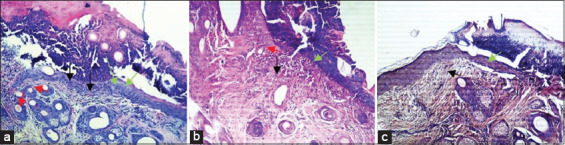
Microscopic view of the different histological aspects of healing, from skin biopsies, performed on the 3^rd^ day after surgery (magnification is 100 ×). Green arrow (reepithelialization), red arrow (neovascularization), black arrow (inflammatory cells). In the group of animals treated with platelet-rich plasma (PRP) (a), the granulation tissue is already developing, with moderate angiogenesis and significant leukocyte infiltration compared to the animals in the group treated with honey (b). Reepithelialization is marked in all wounds, but to varying degrees. The epithelium is hyperplastic in wounds treated with PRP compared to control wounds (c), treated with saline solution. The wound edges appeared to be covered with blood coagulum in all wounds, particularly in the control wounds.

For the other groups, only an accidental infiltration of a few leukocytes in all wounds was observed but without statistical relevance (Figure-2a). Regarding reepithelialization, after 48 h, a layer of epithelium thickened at the free edge of the wound was observed in all groups (Figure-3). In contrast, new blood vessels and newly formed collagen deposits were observed in the groups treated with PRP and honey (0.75±0.45 and 0.33±0.49, respectively) compared with the control group (0±0 and 0±0, respectively). For these parameters, our statistical analysis revealed no significant difference.

**Figure-4:**
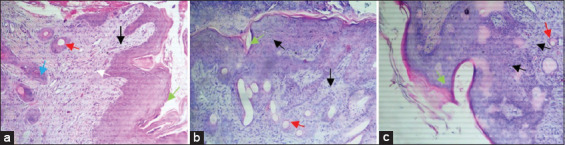
Microscopic view of the different histological aspects of healing, from skin biopsies, taken on the 7^th^ day after surgery (magnification is 100 ×). Green arrow (reepithelialization), red arrow (neovascularization), black arrow (inflammatory cells), blue arrow (macrophages), white arrow (fibroblasts), brown arrow (collagen fibers). In the wounds of the group of animals treated with platelet-rich plasma (a), the epithelial layer showed hypertrophy, with much more differentiated keratinocytes and development of stratum spinosum and stratum granulosum, compared to wounds treated with honey (b) and saline solution (c), respectively.

Seven days after surgery, the inflammatory infiltration phase was completed in all wounds, but without statistical relevance. Histological examination after 7 days revealed the formation of an almost continuous epithelial layer on the incision surfaces in the group of animals treated with PRP with a significant difference (Figure-2b) compared with the control group (1.66±0.49* vs. 0.83±0.57, respectively). Concerning fibroplasia and newly formed blood vessels, these parameters were slightly accelerated in the groups treated with PRP and honey (Figures-4a and 4b) in comparison with the control group (Figure-4c). The number of macrophages and fibroblasts was significantly higher in the PRP-treated group.

**Figure-5:**
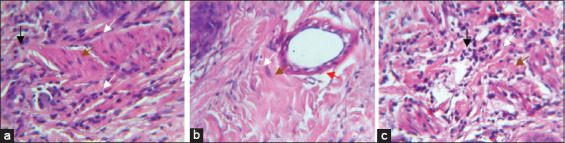
Hematoxylin and eosin stains in wound tissue (a-c) on day 14 after surgery. Black arrow (inflammatory cells), white arrow (fibroblasts), brown arrow (collagen fibers) (magnification is 400 ×). In the dermis of wounds treated with platelet-rich plasma (PRP) (a), collagen fibers much denser, tight, and organized, with an armada of fibroblasts sparse over the entire injured surface. In the dermis of wounds treated with honey (b), the deposition of collagen fibers was less pronounced in comparison with wounds of the PRP group. The samples from the wounds of group (c), treated with the saline solution, showed a dermis strewn with fine collagen fibers and poorly oriented.

Fourteen days after surgery, the synthesis and organization of collagen fibers were significantly improved in the groups treated with PRP and honey (Figure-5a). At an equal rank, the statistical treatment of the results obtained (Figure-2c) revealed a significant difference in favor of wounds treated with honey and a highly significant difference in favor of wounds treated with PRP (2.66±0.49* and 2.75±0.45**, respectively).

**Figure-6:**
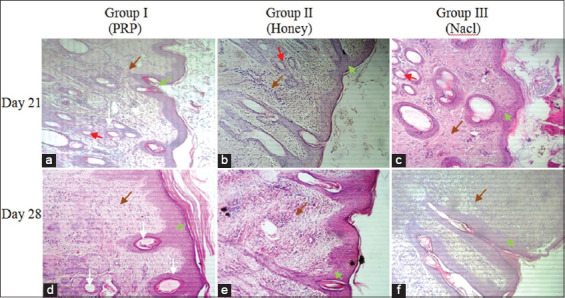
Photomicrographs of various histological appearances (cross section) from skin biopsies taken on the 21^th^ (a-c) and 28^th^ (d-c) days after surgery (magnification is 100×). Green arrow (reepithelialization), red arrow (neovascularization), black arrow (inflammatory cells), white arrow (hair follicle), brown arrow (collagen fibers). On the 21^th^ day of the healing process, all the wounds healed (a-c), with significant variations depending on the different treatments administered. In wounds treated with platelet-rich plasma (PRP) (a), the epidermis was perfectly reconstructed with marked keratinization. The dermis showed well-ordered granulation tissue (dense and oriented collagen fibers) and reappearance of hair follicles. In the group of wounds treated with honey (c), healing of all wounds, but the organization of collagen fibers in the dermis seemed less organized compared to wounds in the PRP group. On the 28^th^ day after surgery, perfect healing of the wounds treated with PRP (d), with excellent reepithelialization, organized dermis exhibits better oriented collagen fibers in comparison with the other groups. Healing was completed in the group treated with honey (e), but the epidermis showed desquamation with poor keratinization. Poor healing of wounds in the control group (f), delayed in most animals treated with saline solution and appearance of pronounced fibrosis at the site of the injury.

Twenty-one days after surgery, reepithelization, fibroplasia, neoangiogenesis, and formation of new bands of collagen were observed and reached their maximum in wounds treated with PRP (Figure-2d). Reepithelialization was significantly improved with completely united tissues in wounds treated with PRP and honey (2.75±0.45 and 2.25±0.62, respectively) compared with that in the control group (1.91±0.79). In contrast, no significant difference in reepithelialization was recorded between wounds treated with PRP and honey (Figure-6).

Twenty-eight days after surgery, all wounds healed. The healing process is especially accelerated in wounds treated with PRP (Figure-2e), characterized by complete reepithelialization with perfect keratinization and reappearance of hair follicles. The collagen fiscal densely filled the wound bed with good reorientation. Delayed poor healing was observed in control wounds with pronounced fibrosis (Figure-6f).

## Discussion

Currently, in the field of regenerative medicine the use of emerging cells therapies, such as PRP, offered a hopeful challenge to clinicians and patients suffering from chronic diseases and resistant to conventional treatments.In contrast, in recent decades, there has been a resurgence in the clinical use of honey as a topical treatment for skin wounds. A great deal of experimental evidence supports this resurgence, showing that honey debrides wounds, kills bacteria, penetrates biofilm, lowers wound pH, reduces chronic inflammation, and promotes fibroblast infiltration [17].

From these results, the topical use of either PRP or honey when managing skin wounds is strongly recommended. Researchers have incorporated honey into tissue engineered models, including electrospun meshes, cryogels, and hydrogels, with varying degrees of success [17,18]. This study was designed to continue the investigation of the biological effects of PRP. The main objective of this study is to compare the main histopathological changes associated with the topical administration of PRP and honey on skin wounds experimentally produced in sheep.

In this study, the wounds were macroscopically and microscopically examined to follow the progress of healing. The semi-quantitative evaluation of the histopathological scores obtained revealed significant differences in several healing parameters.

Three days after surgery, a significant difference in the number of PMNLs was observed with the group of animals treated with PRP having the highest number of PMNLs. At this stage in the healing process, this comparative study showed that honey did not alleviate inflammation compared with PRP. This result agrees with the results of several studies. Anitua *et al*. [19] and Kaux *et al*. [20] have reported that PRP promotes leukocyte diapedesis in the wound bed and accentuates the power of cell differentiation of monocytes into phagocytic ones. Conversely, for honey, we found that it has not reduced inflammation as suggested by Abuharfeil *et al*. [21] and Yaghoobi *et al*. [22]. These authors have reported that honey has absorbent and moisturizing properties that can reduce wound edema and can act on stages of the healing process through early inflammation and rapid proliferation.

Seven days after surgery, in the group of wounds treated with PRP, we recorded significant differences in reepithelialization and fibroblast infiltration. According to Pizzicannella *et al*. [23], these could be due to growth factors released by platelets that stimulate the surrounding cells. This three-dimensional scaffolding provided by PRP is rapidly colonized by fibroblasts, which generate components of the ECM and stimulate the systematic reconstruction of skin tissue [24,25]. This is the proliferative phase, known as the granulation phase. It is marked by significant angiogenesis, accelerated fibroplasia and deposition of ECM, all leading to reepithelialization. The results of this study may lead to a better understanding of the role of PRP as a biostimulator in treating skin wounds [26]. Taken together, PRP represents a positive therapeutic option for damaged cells and tissue regeneration.

Fourteen days after surgery, the synthesis and organization of collagen fibers were significantly improved in the groups of wounds treated with PRP and honey. Our results confirmed the usefulness of the therapeutic use of PRP and honey in managing skin wounds. These are clearly evident in Figure-4a. Nevertheless, despite the large number of studies that confirm the effectiveness of honey in healing skin wounds, in this study, the group of wounds treated with honey was not better than the group treated with PRP. Takzaree *et al*. [27] have reported faster healing and greater reduction of the wound area following the use of honey. In this study, honey promoted the repair of skin wounds in sheep and made the granulation tissue hypertrophic by increasing the synthesis of collagen; however, the latter does not allow the correct course of the remodeling and redesigning processes (Figure-5b).

Twenty-eight days after surgery, healing of all wounds with esthetic variations was quite evident in the micrograph shown in Figures-6d-f. Perfect healing was observed in wounds treated with PRP(Figure-6d), characterized by a similar reconstitution of the skin to normal appearance with well-organized dermis and numerous hair follicles. Our results agree with those of other similar studies [19,25,28]. These studies have reported positive results on the use of PRP as a future cell therapy option for alopecia. According to Miao *et al*. [29] and Xiao *et al*. [28], PRP behaved as an optimal three-dimensional scaffold that induces the cellular expression of versican, beta-catenin, and alkaline phosphatase necessary for hair growth by inducing the differentiation of follicular stem cells of the dermal papilla and prolonging the anagen phase of the hair growth cycle. In addition, the anti-apoptotic effects of activated PRP have been suggested as one of the main factors contributing to the stimulation of hair growth and increase in the perifollicular vascular plexus through increased levels of vascular endothelial growth factor and platelet-derived growth factor, which promote hair growth and angiogenesis [30].

Finally, the results of this experimental study confirm the effectiveness of PRP as a promising remedy in managing skin wounds in sheep. These results should be accepted as preliminary, and the platelet concentration remains uncertain due to interindividual variability and the particular factors influencing each case.

## Conclusion

Wound healing is a continuous and complex process that is characterized by three overlapping phases: The inflammatory, proliferative, and remodeling phases. This process is a series of complex and beautifully orchestrated interactions of cells and mediators, which can be affected by various internal and environmental factors. In this study, topical administration of PRP is a promising method for healing skin wounds in sheep, but the application of honey did not lead to better results. Further in-depth studies to refine a standard preparation protocol for PRP and determine an ideal concentration of PRP to standardize the scientific literature are recommended.

## Authors’ Contributions

DA: Conception and design of the study, realization of the experiment, writing and revision of the manuscript. DO: Data analysis and interpretation, manuscript drafting. Both authors read and approved the final manuscript.
